# Rectal Gas-Induced Dose Changes in Carbon Ion Radiation Therapy for Prostate Cancer: An In Silico Study

**DOI:** 10.1016/j.ijpt.2024.100637

**Published:** 2024-11-26

**Authors:** Miyu Ishizawa, Yuya Miyasaka, Hikaru Souda, Takashi Ono, Hongbo Chai, Hiraku Sato, Takeo Iwai

**Affiliations:** 1 Department of Heavy Particle Medical Science, Yamagata University Graduate School of Medical Science, Yamagata, Japan; 2 Department of Radiation Oncology, Yamagata University Faculty of Medicine, Yamagata, Japan

**Keywords:** Carbon ion radiation therapy, Prostate cancer, Rectal gas, Treatment planning study, Dose evaluation

## Abstract

**Purpose:**

This study aims to determine dosimetric influence of rectal gas in carbon ion radiation therapy (CIRT) for prostate cancer and to establish a procedure for removal rectal gas in clinical scenarios.

**Materials and methods:**

We analyzed 18 prostate cancer cases with bulky rectal gas. The dose distribution was recalculated on computed tomography (CT) with bulky rectal gas (gasCT) after creating the initial plan on a CT without bulky rectal gas, and the doses were transformed using a displacement vector field. This created a dose distribution simulation irradiated with the residual rectal gas. Among 12 fractions (fx) for prostate cancer CIRT, different residual rectal gas fx were used to develop 12 dose distributions, each of which was compared with that in the initial plan. Clinical target volume (D_min_, D_99.5%_), rectum, and rectal wall (V_95%_, V_80%_) parameters were assessed. We investigated the indicators associated with these dose changes using digital reconstruction radiograph (DRR) images.

**Results:**

The dosimetric changes in the clinical target volume were not significantly different from that in the initial treatment plan for both D_min_ and D99.5%. Compared to the initial plan, the dose-volume histogram parameters showed changes exceeding 1 cm^3^ when residual rectal gas was present in the following number of fractions: 8 fx for V_95%_ rectum, 5 fx for V_80%_ rectum, 10 fx for V_95%_ rectal wall, and 11 fx for V_80%_ rectal wall. Changes in rectal and rectal wall parameters were highly correlated with the extent of rectal gas assessed on DRR images.

**Conclusion:**

Rectal gas removal may not be necessary up to 4 fx. Moreover, indicators related to dose changes based on DRR images were highly correlated with dose changes, revealing the possibilities of estimating dose changes due to rectal gas from kV-x-ray images and using gas effect evaluation during CIRT irradiation.

## Highlights

• Rectal gases may affect dose distribution for prostate cancer in carbon ion therapy.
• As the impact is unclear, removing bulky rectal gas was mandatory at our institution.
• We evaluated dose changes using clinical CT and CT imaging with bulky rectal gas.
• Even with rectal gas present for up to 4 of 12 fractions, dose change remained small.

## Introduction

Prostate cancer exhibits the second highest incidence (14.1%) and fifth highest mortality rates (6.8%) in males.^1^ Radiation therapy is an effective radical treatment for localized prostate cancer.^2^ Recent clinical studies have verified the efficacy of proton beam therapy and carbon ion radiation therapy (CIRT).^3^, ^4^, ^5^, ^6^, ^7^, ^8^, ^9^

CIRT demonstrates superior physical and biological advantages compared with conventional radiation therapy with x-rays. The physical advantage includes a better dose distribution using sharp Bragg peaks and a small lateral penumbra, and the biological advantage is the relative biological effectiveness (RBE) value of carbon ion beams of approximately 3 due to the higher linear energy transfer.^10^, ^11^ This enables dose escalation while significantly reducing the dose in the organ at risk (OAR) in normal tissue. The fractionation schemes in CIRT for prostate cancer started with a protocol that administered 60 to 66 Gy/20 fractions (fx).^12^ Next, to improve efficacy and safety, it was administered at 57.6 Gy/16 fx, which is equivalent to administering 63 Gy/20 fx.^13^ Adverse events were reduced to <10% with this number of fractions. Furthermore, encouraged by the aforementioned benefits, a protocol of 51.2 Gy/12 fx was developed.^14^ In Japan, this fractionation scheme is common, and sufficient results have been reported in terms of treatment outcomes and adverse events.^6^, ^15^, ^16^ Therefore, this dose fractionation is used at our hospital.

However, the dose distribution of particle therapy is sensitive to anatomical heterogeneities in the patient along the beam path.^17^, ^18^ Rectal gas alters internal states between treatment planning and irradiation and affects the dose distribution.^19^ To reduce the effect of rectal gas, considering uncertainty in treatment planning and controlling the patient’s rectum are crucial. One method of considering uncertainty in treatment planning is robust optimization.^20^ Controlling the patient’s rectal positioning includes placement of a rectal balloon,^21^ administration of laxatives or herbal remedies and enemas,^22^, ^23^ and schemes of drinking and elimination performance.^24^ These measures need to be determined following the degree of gas influence in actual CIRT. However, to the best of our knowledge, no studies have quantitatively analyzed the effect of these measures in reducing dose change in CIRT; hence, whenever rectal gas is present in the patient on the treatment couch during onboard pretreatment imaging, rectal gas is removed in some CIRT centers, including our center.

Rectal gas removal is a burdensome process for both patients and medical staff. Patients often experience discomfort and embarrassment due to the invasive nature of the procedure. Medical staff must remove the immobilization device and reposition the patient on their side to carry out the process, which requires repeating image-guided radiation therapy (IGRT). Although the procedure itself takes approximately 5 minutes, it reduces the overall efficiency of the treatment room due to the need to repeat IGRT. Limiting rectal gas removal is important for patients, and the medical staff, and pretreatment images for positioning are useful to assess rectal gas removal. A 3-dimensional image-guided system enables easy checking of organ movement, including changes due to rectal gas. However, the reduction in throughput caused by the additional couch shift time and increased imaging time may lead to organ motion and positional shifts. Therefore, rapid assessment using a two-dimensional (2D) image-guided system is necessary; however, no study has investigated the association between readable indicators that reflect rectal deformation due to rectal gas from a 2D image-guided system and dose change. Our hospital also uses a 2D image-guided system for IGRT, but the decision to remove rectal gas is made by the radiation oncologist or radiation technologist, and there are no clinical standards in place.

The final goal is to understand the dosimetric impact of rectal gas and to develop clinical criteria for its removal. This study serves as an initial step toward achieving that goal. More specifically, this study aimed to quantitatively assess the dose change in CIRT for prostate cancer when irradiated with bulky rectal gas as well as determine indicators related to dose changes in target and OAR when 2-dimensional kilovoltage (kV)-x-ray flat-panel detector images are used for IGRT.

## Material and methods

### Patient selection

This study selected 20 patients who had undergone CIRT for prostate cancer from April 2023 to October 2023 with at least 2 treatment planning computed tomography (pCT) data sets with or without bulky rectal gas. A radiation technologist collected the pCT, and if there was substantial rectal gas or stool, another pCT was performed after the rectal gas was removed. This occurred at the same appointment. This study analyzed 18 of 20 patients who were scanned twice, excluding 2 patients who showed substantial stool even after rescanning. Our institutional research ethics committee approved this study.

### Treatment planning computed tomography and contouring

An Aquilion ONE (Canon Medical Systems, Otawara, Japan) was used to perform pCT, with a 2-mm CT slice thickness and 120-kV voltage. All patients were immobilized in the supine position using thermoplastic shells (HipFix, CIVCO Radiotherapy) and vacuum bags (Blue Bag, Elekta AB). Two CT images were used, including the pCT in clinical practice and a CT with residual bulky rectal gas (gasCT), which was indicated as unacceptable for CIRT by the radiation oncologist. Briefly, the pCT was clinical and the gasCT was the first scan that was not used for planning due to gas. All patients were identified as medium-risk based on the D′Amico risk classifications.^25^ The clinical target volume (CTV) included the prostate and base of the seminal vesicles. The delineation of the rectum as an OAR stretched from 14 mm superior to the upper margin of the CTV to 14 mm inferior to its lower margin. The rectal wall was created from the rectum as an inner 2-mm ring structure. We do not use SpaceOAR for prostate cancer in CIRT in clinical practice, and all of the cases in this study were patients who had not used it. An experienced radiation oncologist defined all regions of interest (ROIs), and the planning target volume (PTV) included margins in the right, left, and anterior directions. MIM Maestro software version 5.6. (MIM Software Inc) was used to construct all targets and OARs.

### Treatment planning

The parameters of the initial treatment plan for carbon-ion pencil beam scanning were calculated using the pCT, with a prescribed RBE-weighted dose of 51.6 Gy/12 fx (median dose prescription).^6^, ^26^, ^27^ We used the lateral opposite fields (6 fx each). The optimization objectives for all treatment plans were similar, and the single-field uniform dose method was utilized to meet the dose constraints shown in Table 1.

**Table 1:** Dose constraints.

Structure	Constraint
CTV	D_min_ > 47.90 Gy
	D_99.5%_ > 49.02 Gy
Rectum	V_95%_ < 0.80 cm^3^
	V_80%_ < 4.10 cm^3^
Rectum wall	V_95%_ < 0.80 cm^3^
	V_80%_ < 4.10 cm^3^

**Abbreviations:** CTV, clinical target volume; D_min_, minimum dose; V_x%_, the volume irradiated by x% or more of the prescribed dose; D_y%_, the dose irradiated by y% or more of the volume.

Conditional robust optimization of 2 mm in the anterior-posterior and superior-inferior (SI) directions was adapted to the PTV.^28^ RayStation 10A (RaySearch Laboratories AB), using a pencil beam algorithm with a calculation grid size of 2 mm × 2 mm × 2 mm, was used for all dose calculations. A pCT with rectal gas was replaced using a stopping power ratio of water. The modified microdosimetric kinetic model was used for biological dose calculations.^29^, ^30^, ^31^ The modified microdosimetric kinetic model assumed that cell survival in subcellular volumes (domains) followed a linear-quadratic model when exposed to specific energy and used the Kiefer-Chatterjee track structure model to modify regarding saturation of RBE. The cell type was HSG, and the radii of the cell nucleus and the domain $r_{n}$, $r_{d}$ were 0.320 µm and 3.900 µm, respectively. The values of $\alpha_{0}$ and β, which indicated the cell survival fraction, were set to 0.1720/Gy and 0.0615/Gy, respectively. $z_{1D}^{*}$ indicated dose-weighted mean specific energy of the domain.

### Dose simulation in the presence of bulky rectal gas

**Figure 1:**
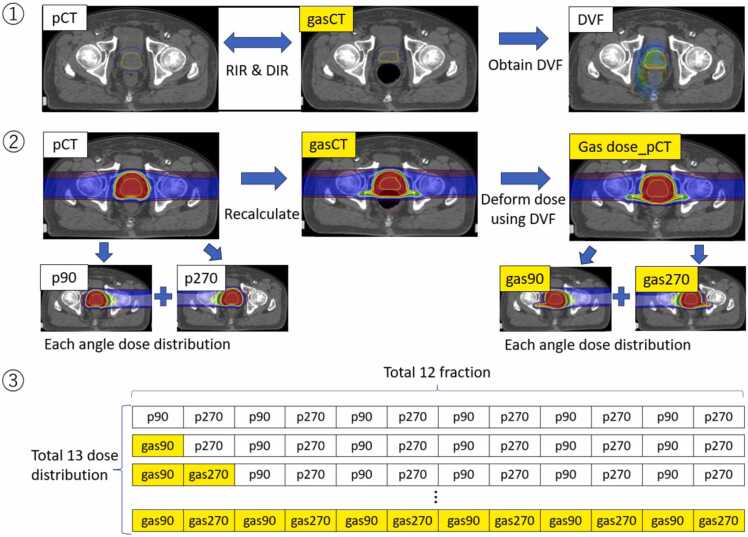
Evaluation workflow. First, RIR and DIR were applied between pCT and gasCT, and DVF was obtained. Second, the initial treatment plan on pCT was recalculated on gasCT based on RIR, and DVF was used to deform the dose distributions on pCT. Third, per-fraction dose distributions were established for the beams from 90° to 270° on the pCT and gasCT (p90, p270, gas90, and gas270, respectively), which were summed together, totaling 12 fx. Abbreviations: DIR, deformable rigid registration; DVF, displacement vector field; gasCT, CT of remaining high rectal gas; pCT, treatment planning CT; and RIR, rigid registration.

Figure 1 shows the dose simulation method in the presence of bulky rectal gas. First, a rigid registration (RIR) and a deformable image registration (DIR) were applied between the pCT and the gasCT, and a displacement vector field (DVF) was obtained. RIR was performed as bone matching in 6 axes, including translation and rotation. DIR was performed using a hybrid intensity and structure-based algorithm via RayStation.^32^ Second, the initial treatment plan on the pCT was recalculated on the gasCT based on the RIR with all beam parameters fixed, and a DVF was used to deform these dose distributions on pCT. In other words, the dose distribution was distorted using the derived DVF based on DIR between the pCT and the gas CT rather than simply recalculating the dose. Rectal gas caused the rectum to expand, altering its position relative to the target and changing the volume and position of the rectal wall within the beam path. Therefore, this method was chosen to evaluate the dose in the same anatomical region of the rectal wall as accurately as possible. The resulting dose distribution was named “gas dose_pCT.” Third, per-fraction dose distributions were established for the 4 beams from 90° (left) and 270° (right) on pCT and gasCT (p90, p270, gas90, and gas270, respectively) and were summed together in 12 fx. The gasCT dose distributions were increased by 1 fx, starting from gas90, establishing a total of 13 dose distributions with different fractions. We used the Hausdorff distance and Dice coefficient to assess the DIR accuracy of CTV and rectum.^33^, ^34^ The dose-volume histogram (DVH) parameters, as shown in Table 1, were calculated for hypothetical dose distributions with the number of rectal gas residuals of 1 to 12 fx. We then determined the difference in the DVH parameters of each dose distribution compared with the initial treatment plan. All DVH parameters used in this study were adopted from those applied in our clinical practice. For CTV, we used the lowest dose and a value close to it, prioritizing the fact that the dose covered the target more strictly than the commonly evaluated D_95%_. The rectal V_95%_ was 49.02 Gy, which was nearly identical to other studies that evaluated it at V_50 Gy_.^4^ The rectal V_80%_ was also referenced in other reports.^4^, ^35^ We selected this parameter because we believed the rectal wall could be evaluated for dose without being influenced by rectal deformation caused by rectal gas.

### Evaluation of dose change-related indicators based on digital reconstruction radiograph images

**Figure 2:**
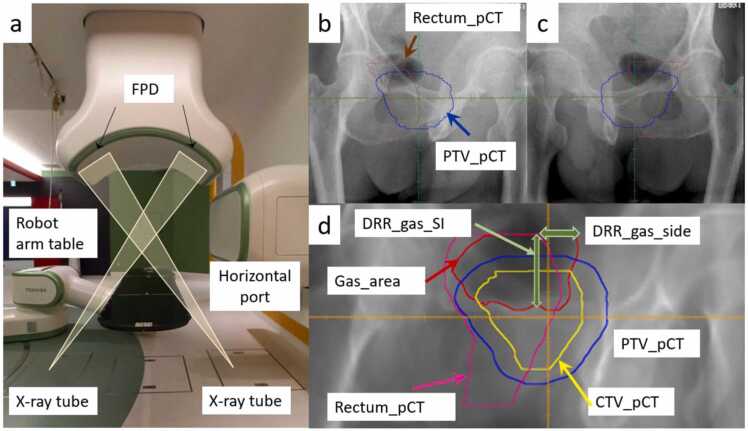
Images to complement the method. (a) Arrangement of an irradiation room, oblique kV-x-ray image system; (b), (c) kV-x-ray images (35° and 325°, respectively); (d) defined indicator on digital reconstruction radiograph. Abbreviations: DRR, digital reconstruction radiograph; pCT, treatment planning CT; PTV, planning target volume; and SI, superior-inferior.

Digital reconstruction radiograph (DRR) images from 2 oblique directions (35° and 325°) were evaluated, as these were the only directions available for kV x-ray imaging in our hospital, as shown in Figure 2a. Figure 2b and c shows the kV-x-ray images. Figure 2d presents the definition of dose change-related indicators. First, the “CTV_pCT,” “PTV_pCT,” and “Rectum_pCT” (CTV, PTV, and Rectum of ROI on pCT, respectively) were displayed on the DRR of the gasCT. Second, a “Gas_area” ROI was created on the DRR as an area with a Hounsfield Unit of −1200 to −120 in Rectum_gasCT. Third, “DRR_gas_SI,” “DRR_gas_side,” and “DRR_gas_area” were defined based on the 4 ROIs as the distance between gas area and PTV_pCT in the SI direction, the distance of Gas_area in PTV_pCT that extended out from Rectum_pCT, the product of DRR_gas_SI and DRR_gas_side, respectively. Pearson’s correlation coefficients between the DVH parameters, as shown in Table 1, and DRR_gas_side, DRR_gas_SI, and DRR_gas_area were investigated using MATLAB R2023a (MathWorks, Natick). The correlation coefficients between rectal volume and rectal gas volume were calculated for comparison. The change in the rectum and rectal wall parameters was calculated in terms of absolute volume change.

### Statistical analysis

MATLAB was used for statistical analysis. All nonparametric data were compared using Wilcoxon signed-rank tests to evaluate significant differences regardless of whether they followed a normal distribution because of the sample size (18 cases). *P*-values of <.05 were considered to indicate statistical significance.

## Results

### Evaluation of rectal gas volume and deformable image registration

The mean ± standard deviation values of rectal gas volume for the pCT and the gasCT were 0.83 ± 1.63 and 17.54 ± 11.10 cm^3^, respectively. For the CTV and rectum, the mean Hausdorff distances were 4.33 ± 1.52 and 3.32 ± 3.35 mm, and the mean Dice coefficients were 0.98 ± 0.01 and 0.98 ± 0.02, respectively.

### Changes in dose-volume histogram parameters due to residual rectal gas

**Figure 3:**
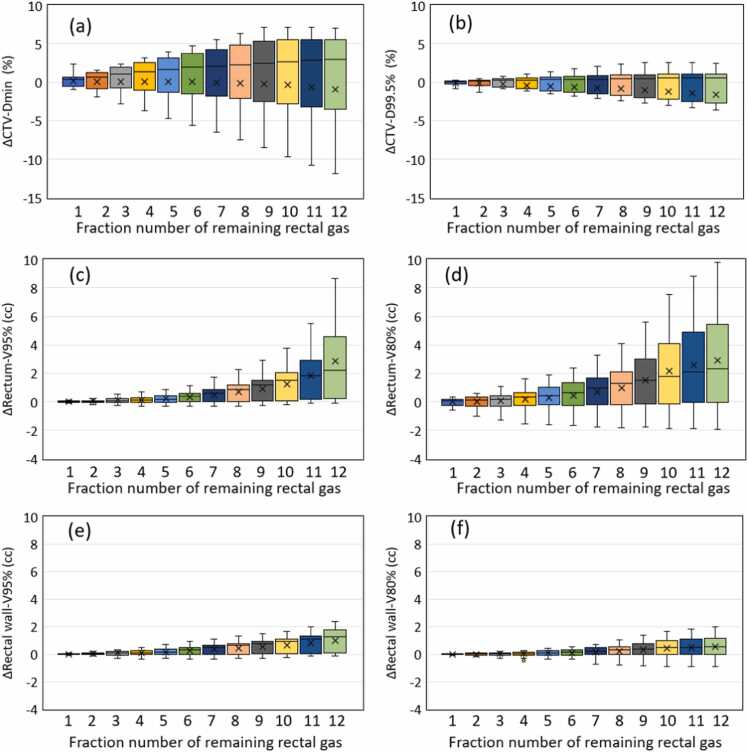
Box-and-whisker plots of changes in DVH parameters compared with the initial treatment plan for varying the number of rectal gas residuals. (a) and (b) D_min_ and D_99.5%_ of CTV. (c) and (d) V_95%_ and V_80%_ of the rectum. (e) and (f) V_95%_ and V_80%_ of the rectal wall. Abbreviations: CTV, clinical target volume; D_min_, minimum dose; D_y%_, the dose irradiated by y% or more of the volume; and V_x%_, the volume irradiated by x% or more of the prescribed dose.

Supplementary Table 1 shows the DVH parameters for the initial treatment plan (0 fx) and each DVH parameter when simulating the number of rectal gas residues from 1 fx to 12 fx. For example, in the case of "3 fx," the DVH parameters are shown for a RayStation simulation where 3 fractions out of 12 fractions were irradiated with residual rectal gas, and the remaining 9 fractions were not. Additionally, Figure 3 shows the changes in the DVH parameter from 0 fx. Regarding CTV, assuming residual gas in all 12 fractions and evaluating the data using the interquartile range, D_min_ changed by ±5.6% and D_99.5%_ by ±2.7% compared to the initial plan. Even when simulating with residual gas in all fractions, there was no significant difference between the initial plan and the CTV in either D_min_ or D_99.5%_, which are indicators of coverage (*P* = .794 [12 fx] and *P* = .420 [12 fx], respectively). Conversely, V_95%_ rectum and V_95%_ rectal wall were significantly different at 4 fx (*P* = .037 and *P* = .039, respectively), and V_80%_ rectum and V_80%_ rectal wall were significantly different at both 8 and 7 fx (*P* = .028 and *P* = .035, respectively). Compared to the initial plan, the DVH parameters showed changes exceeding 1 cm^3^ when residual rectal gas was present in the following number of fractions: 8 fx for V_95%_ rectum, 5 fx for V_80%_ rectum, 10 fx for V_95%_ rectal wall, and 11 fx for V_80%_ rectal wall, evaluated at the 75th percentile. Similarly, the numbers of rectal gas residuals for a fraction of >2 cm^3^ from the initial plan for V_95%_ rectum and V_80%_ rectum were 10 and 8 fx, respectively. V_95%_ and V_80%_ rectal wall did not exceed 2 cm^3^ (Figure 3).

**Figure 4:**
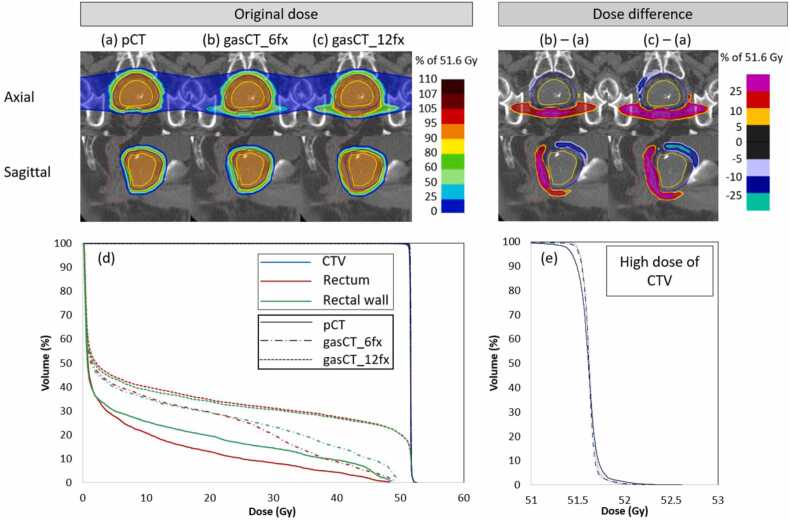
Dose distribution and dose-volume histograms of original dose and dose difference. Patient 15 with the largest dose changes in the rectum and rectal wall. (a)-(c) The initial treatment plan dose (pCT), dose distribution of 6 fx residual rectal gas (gasCT_6 fx), and gasCT_12 fx; (b)-(a) and (c)-(a) dose differences compared with (a) pCT; (d) dose-volume histograms of (a), (b), and (c); (e) high dose areas of (d) only CTV. Abbreviations: CTV, clinical target volume; gasCT, CT of remaining high rectal gas.

Figure 4 shows the dose distribution of the initial treatment plan for patient 15, who had the largest dose change in the rectum and rectal wall, and the dose distribution and DVH parameters for the 6 fx and 12 fx residual rectal gas. The rectum, in contact with the PTV, had a higher dose, whereas The superior and anterior sides of the PTV had slightly lower doses.

### Evaluation of dose change-related indicators based on digital reconstruction radiograph

Table 2 shows the correlation coefficients between indicators and the DVH parameters. All DVH parameters were used for all 12 fx of residual rectal gas. The indicator with the highest correlation coefficient with CTV was DRR_gas_side. The indicator with the highest correlation coefficient with CTV was DRR_gas_side (0.554 and 0.442 for D_min_ and D_99.5%_, respectively), whereas that with the highest correlation coefficient with the rectum and rectal wall was DRR_gas_area (rectum: 0.632, 0.570, and 0.536 and rectal wall: 0.605, 0.674, and 0.660 for D_95%_, D_80%_, and D_50%_, respectively).

**Table 2:** Correlation coefficients between each DVH parameter and various indicators.

Structure	ΔDVH parameter	ΔRectum volume (cm^3^)	ΔRectum gas volume (cm^3^)	DRR_gas_side (cm)	DRR_gas_SI (cm)	DRR_gas_area (cm^2^)
CTV	ΔD_min_ (%)	0.137	0.213	0.544^a^	0.504	0.464
	ΔD_99.5%_ (%)	0.239	0.218	0.442^a^	0.426	0.411
Rectum	ΔV_95%_ (cm^3^)	0.479	0.570	0.444	0.578	0.632^a^
	ΔV_80%_ (cm^3^)	0.397	0.459	0.437	0.524	0.570^a^
Rectal wall	ΔV_95%_ (cm^3^)	0.453	0.432	0.429	0.584	0.605^a^
	ΔV_80%_ (cm^3^)	0.380	0.400	0.520	0.655	0.674^a^

**Abbreviations:** CTV, clinical target volume; D_min_, minimum dose; V_x%_, the volume irradiated by x% or more of the prescribed dose; D_y%_, the dose irradiated by y% or more of the volume.

DRR_gas_SI: distance between gas area and PTV_pCT in the superior-inferior (SI) direction; DRR_gas_side: distance of “Gas_area” in PTV_pCT that extended out from Rectum_pCT; Gas_area: ROI on the DRR as an area with Hounsfield Unit ranging from −1200 to −120 in Rectum_gasCT; DRR_gas_area: product of DRR_gas_SI and DRR_gas_side.

The correlation coefficients between the change from pCT and the 5 parameters when all 12 fr were gas. The DRR_gas_side parameter correlated best with the CTV dose change. The DRR_gas_area parameter correlated best with the rectum and rectal wall dose change.

a Indicators with the highest correlation coefficient for each ΔDVH parameter.

## Discussion

We investigated the association of rectal gas with the CIRT dose for prostate cancer. D_min_ ranged from −3.6% to 5.6%, and D_99.5%_ ranged from −2.7% to 1.0% for CTV at 12 fx. This finding indicates that rectal gas does not cause unacceptable underdose in the CTV. The need for rectal gas removal to avoid degraded dose distribution was considered less important in our treatment planning. Conversely, CTV dose changes may vary depending on treatment planning performance. The DVH parameter of CTV may decrease to a greater extent if no robust optimization is used or the PTV margin is smaller than that in our treatment planning.

A previous study reported that rectal gas is more likely to occur in the same location.^36^ The rectal gas in the 18 cases analyzed in this study was mainly located superior to the CTV and rectum (Supplementary Figure 1). This was consistent with several other studies indicating that the upper part of the rectum is more anatomically fixed.^36^, ^37^, ^38^, ^39^, ^40^ This area contains the sagittal plane where the base of the seminal vesicle is located; thus, the dose change is expected to be greater where the CTV contains more seminal vesicles. Examining dose changes that target a large part of the seminal vesicle separately is necessary because the results of the current study were based on the assumption of medium-risk CTV, which was defined as covering the whole prostate and base of the seminal vesicle.

Late gastrointestinal (GI) toxicity after radiation therapy for prostate cancer is an important late adverse event, and studies have reported that the rectum dose was strongly correlated with late rectal hemorrhage.^41^, ^42^, ^43^ The V_95%_ and V_80%_ rectum exceeded the dose constraints from 5 and 4 fx, respectively, when evaluated with the dose constraints shown in Table 1. The dose constraints used in the current study were more stringent than those used in other studies.^6^, ^14^, ^35^ In CIRT for prostate cancer of 12 fx, Sato et al^6^ used V_53 Gy_ of <0%, V_50 Gy_ of <7%, and V_40 Gy_ of <16% as recommended dose constraints in the rectum. Approximate conversion using this dose constraint revealed that the V_95%_ and V_80%_ rectum exceeded the dose constraints from 10 fx. Sato et al^6^ revealed no acute GI rectal toxicity, and the incidences of grades 2 and 3 of late GI toxicity were 0.4% and 0.0%, respectively.^6^ A study analyzing the toxicity of photon IMRT revealed a 5.8% incidence of late GI toxicity,^44^ and a phase II clinical trial of proton therapy demonstrated a 13% incidence of late GI toxicity.^45^ The incidences of acute-phase and late GI toxicities reported by Sato et al^6^ were considerably lower than those reported following photon and proton therapy, indicating that this is a sufficiently safe rectal constraint. Therefore, up to 4 fx of residual rectal gas can be treated without an increase in the incidence of late GI toxicity and without significant deviation from the initial treatment plan in terms of dose increase and dose constraints. This finding depends on the treatment planning method used in the study. Shin et al^46^ revealed that dose distribution using oblique irradiation fields (60°-300° and 120°-240°) was superior in dose preservation to the rectum compared with dose distribution using conventional side-opposed fields (90°-270°) for prostate cancer. Oblique irradiation fields may become more predominant with the continuous development of gantry CIRT systems. However, most facilities currently use the horizontal irradiation method for prostate cancer. Thus, the results of this study are considered useful. In addition, this study uses a single RBE model for analysis. However, there is a report on converting MKM to LEM doses,^47^ which indicates that the conversion factor for 4.3 Gy/fx used in this study was approximately 1.08. (The conversion coefficients for MKM doses of 4.20 and 4.38 Gy per fraction are 1.09 and 1.07, respectively, and the coefficient for 4.3 Gy was calculated using linear interpolation.) Therefore, it is possible to convert from MKM to LEM using this value, and we believe that the results of this study will also be applicable to LEM.

kV-x-ray images are used for IGRT by matching the bones and are widely utilized in most facilities that perform CIRT.^48^, ^49^ There are 2 types of kV-x-ray images: frontal and lateral (0 degrees and 90 degrees) and oblique (35 degrees and 225 degrees). Currently, frontal and lateral images are the most common; however, our hospital can only use oblique images. It is anticipated that if the irradiation systems of our hospital become more widespread in the future, the number of oblique kV-x-ray images will increase. The method of estimating dose changes due to rectal gas from kV-x-ray images and using gas effect evaluation during CIRT irradiation might be useful at other facilities as well. The indicator with the highest correlation coefficient with CTV was DRR_gas_side, and that with the highest correlation coefficient with the rectum and rectal wall was DRR_gas_area. This is because DRR_gas_area was considered an indicator of both DRR_gas_side and DRR_gas_SI. Conversely, rectal gas for CTV causes the CTV to tilt ventrally, leading to the appearance of a cold spot at the superior of the CTV (Supplementary Figure 1). This may be due to the amount of rectal gas present in the anterior-posterior direction rather than the SI direction; therefore, the correlation coefficient of CTV could be the highest for DRR_gas_side rather than for gas DRR_gas_area. These indicators are easily measured on a monitor for positioning without any other device. Moreover, this facilitates not only the investigation of the correlation coefficient between these indicators and the DVH parameter but also the accurate prediction of the change in the DVH parameter, which can be used clinically.

This study had several limitations. First, each patient had only 1 gasCT, and the results of dose variation with different numbers of rectal gas residuals are based on the assumption that the state of rectal gas is similar each time. To evaluate the reproducibility of the amount and location of rectal gas, we analyzed a separate data set from this study (Supplementary material). The variation in rectal gas volume over 5 fractions averaged 2.9 cm^3^ within patients. The location of rectal gas was cranial or both cranial and middle in approximately 95% of cases, and the difference in position within 5 fractions was within 5 mm. Based on these findings, it might be said that there is a certain degree of reproducibility in both the volume and location of rectal gas within patients. Therefore, even with only 1 gas CT evaluation, clinically useful information might be obtained without significant misinterpretation. However, it should be noted that the volume and location of rectal gas are not always consistent, and in such cases, the distribution of errors could lead to an overestimation of the results in this study. Artificial intelligence and image processing may reveal more quantitative and correlative parameters. Second, an additional beam from 90° was considered in the case of an odd number of rectal gas residuals, and the same numbers of beams from 90° and 270° were considered in the case of an even number of rectal gas residuals. In some cases, the prostate was tilted to the left or right or rectal distension was observed to the right or left; thus, the results may vary slightly depending on the type of beam used. However, our results will remain consistent because most cases demonstrated no left-right differences. The third limitation is that clinical data could not be included in the analysis, and this study remains a basic investigation in silico. Including clinical data would likely make the conclusions and recommendations of this study more justifiable. However, we believe that understanding approximate dose changes is useful in supporting the judgment of clinical medical staff.

A future consideration is to construct a system that automatically determines the need for rectal gas removal using these reports.^50^, ^51^ Specifically, it is a system that can accurately identify the position of the rectum and the position of the rectal gas by simply scanning kV images and evaluating and determining the impact on the dose based on the degree of change from the treatment plan.

## Conclusion

This study revealed the dose changes in CTV, rectum, and rectal wall in prostate cancer CIRT when irradiated with residual rectal gas based on the treatment plan. A dose accuracy of within 5% is required for successful radiation therapy,^52^ even when rectal gas was present in all 12 fx, the D_99.5%_ of the CTV did not change by more than 5%. Changes from the initial plan concerning the rectum V_80%_ exceeding 1 cm^3^ occurred when residual rectal gas was present at 5 fx. The results indicated rectal gas removal may not be necessary up to 4 fx. We also revealed indicators related to dose changes based on DRR images and demonstrated that these indicators were highly correlated with dose changes. This finding reveals the possibility of estimating the dose change due to rectal gas from kV-x-ray images and using gas effect evaluation during CIRT irradiation.

## Ethics

The Research Ethics Committee of the Yamagata University approved this study (2023-144).

## Funding

This research did not receive any specific grant from funding agencies in the public, commercial, or not-for-profit sectors.

## Author Contributions

Miyu Ishizawa: Methodology, Data curation, Writing- Original draft, Statistical analyses, Writing- Review and Editing. Yuya Miyasaka: Conception, Formal analysis, Writing- Review and Editing. Takashi Ono: Methodology, Writing- Review and Editing. Hikaru Souda, Hongbo Chai, Hiraku Sato, Takeo Iwai: Writing- Review and Editing.

## Declaration of Conflicts of Interest

The authors declare that they have no known competing financial interests or personal relationships that could have appeared to influence the work reported in this paper.

## Disclosure of the Use of Generative AI

During the preparation of this work, the author used DeepL for only to improve language and readability. After using this tool/service, the author reviewed and edited the content as necessary and is fully responsible for the content of the publication.

## Data Availability

Research data are stored in an institutional repository and will be shared upon request to the corresponding author.

## Appendix A Supplementary material

Supplementary Figure 1 Rectal gas location. Abbreviations: gasCT: CT of remaining bulky rectal gas. (a), (b), (c), (d), (e), and (f) show the sagittal plane of the gasCT for patients 1, 3, 4, 11, 12, and 17, respectively. CTV is outlined in yellow, PTV in dark blue, and rectum in brown. Rectal gas was mainly located superior to the CTV and rectum.

## Footnotes

Appendix A Supplementary data associated with this article can be found in the online version at doi:10.1016/j.ijpt.2024.100637.

## References

[1] Sung H., Ferlay J., Siegel R.L. (2021). Global cancer statistics 2020: GLOBOCAN estimates of incidence and mortality worldwide for 36 cancers in 185 countries. CA Cancer J Clin.

[2] Mason M.D., Parulekar W.R., Sydes M.R. (2015). Final report of the intergroup randomized study of combined androgen-deprivation therapy plus radiotherapy versus androgen-deprivation therapy alone in locally advanced prostate cancer. J Clin Oncol.

[3] Kawamura H., Kubo N., Sato H. (2020). Moderately hypofractionated carbon ion radiotherapy for prostate cancer; a prospective observational study “GUNMA0702”. BMC Cancer.

[4] Nomiya T., Tsuji H., Kawamura H. (2016). A multi-institutional analysis of prospective studies of carbon ion radiotherapy for prostate cancer: a report from the Japan Carbon ion Radiation Oncology Study Group (J-CROS). Radiother Oncol.

[5] Takakusagi Y., Katoh H., Kano K. (2020). Preliminary result of carbon-ion radiotherapy using the spot scanning method for prostate cancer. Radiat Oncol.

[6] Sato H., Kasuya G., Ishikawa H. (2021). Long-term clinical outcomes after 12-fractionated carbon-ion radiotherapy for localized prostate cancer. Cancer Sci.

[7] Grewal A.S., Schonewolf C., Min E.J. (2019). Four-year outcomes from a prospective phase II clinical trial of moderately hypofractionated proton therapy for localized prostate cancer. Int J Radiat Oncol Biol Phys.

[8] Kubeš J., Sláviková S., Vítek P. (2023). 5-Years analysis of effectivity and toxicity of ultra-hypofractionated proton radiotherapy in the treatment of low- and intermediate-risk prostate cancer—a retrospective analysis. Cancers.

[9] Bryant C.M., Hoppe B.S. (2021). Promising long-term results with proton therapy for localized prostate cancer. Nat Rev Urol.

[10] Kanai T., Matsufuji N., Miyamoto T. (2006). Examination of GyE system for HIMAC carbon therapy. Int J Radiat Oncol Biol Phys.

[11] Fowler J.F. (2005). The radiobiology of prostate cancer including new aspects of fractionated radiotherapy. Acta Oncol.

[12] Ishikawa H., Tsuji H., Kamada T. (2012). Carbon-ion radiation therapy for prostate cancer. Int J Urol.

[13] Okada T., Tsuji H., Kamada T. (2012). Carbon ion radiotherapy in advanced hypofractionated regimens for prostate cancer: from 20 to 16 fractions. Int J Radiat Oncol Biol Phys.

[14] Nomiya T., Tsuji H., Maruyama K. (2014). Phase I/II trial of definitive carbon ion radiotherapy for prostate cancer: evaluation of shortening of treatment period to 3 weeks. Br J Cancer.

[15] Ishikawa H., Hiroshima Y., Kanematsu N. (2022). Carbon-ion radiotherapy for urological cancers. Int J Urol.

[16] Takakusagi Y., Koge H., Kano K. (2024). Five-year clinical outcomes of scanning carbon-ion radiotherapy for prostate cancer. PLoS One.

[17] Urie M., Goitein M., Holley W.R., Chen G.T. (1986). Degradation of the Bragg peak due to inhomogeneities. Phys Med Biol.

[18] Sawakuchi G.O., Titt U., Mirkovic D., Mohan R. (2008). Density heterogeneities and the influence of multiple Coulomb and nuclear scatterings on the Bragg peak distal edge of proton therapy beams. Phys Med Biol.

[19] Soukup M., Söhn M., Yan D. (2009). Study of robustness of IMPT and IMRT for prostate cancer against organ movement. Int J Radiat Oncol Biol Phys.

[20] Butala A.A., Ingram W.S., O'Reilly S.E. (2020). Robust treatment planning in whole pelvis pencil beam scanning proton therapy for prostate cancer. Med Dosim.

[21] Wachter S., Gerstner N., Dorner D. (2002). The influence of a rectal balloon tube as internal immobilization device on variations of volumes and dose-volume histograms during treatment course of conformal radiotherapy for prostate cancer. Int J Radiat Oncol Biol Phys.

[22] Tsuji H., Yanagi T., Ishikawa H. (2005). Hypofractionated radiotherapy with carbon ion beams for prostate cancer. Int J Radiat Oncol Biol Phys.

[23] Nagai A., Shibamoto Y., Ogawa K., Inoda K., Yoshida M., Kikuchi Y. (2016). Analysis and management of rectal gas with kampo formulas during intensity-modulated radiotherapy of prostate cancer: a case series study. J Integr Complement Med.

[24] Fuchs F., Habl G., Devečka M. (2019). Interfraction variation and dosimetric changes during image-guided radiation therapy in prostate cancer patients. Radiat Oncol J.

[25] Zhao J., Mangarova D.B., Brangsch J. (2020). Correlation between intraprostatic PSMA uptake and MRI PI-RADS of [68Ga]Ga-PSMA-11 PET/MRI in patients with prostate cancer: comparison of PI-RADS version 2.0 and PI-RADS version 2.1. Cancers.

[26] Furukawa T., Inaniwa T., Sato S. (2010). Performance of the NIRS fast scanning system for heavy-ion radiotherapy. Med Phys.

[27] Minohara S., Fukuda S., Kanematsu N. (2010). Recent innovations in carbon-ion radiotherapy. J Radiat Res.

[28] Fredriksson A., Forsgren A., Hårdemark B. (2011). Minimax optimization for handling range and setup uncertainties in proton therapy. Med Phys.

[29] Kase Y., Kanai T., Matsumoto Y. (2006). Microdosimetric measurements and estimation of human cell survival for heavy-ion beams. Radiat Res.

[30] Hawkins R.B. (1996). A microdosimetric-kinetic model of cell death from exposure to ionizing radiation of any LET, with experimental and clinical applications. Int J Radiat Biol.

[31] Inaniwa T., Furukawa T., Kase Y. (2010). Treatment planning for a scanned carbon beam with a modified microdosimetric kinetic model. Phys Med Biol.

[32] Weistrand O., Svensson S. (2015). The ANACONDA algorithm for deformable image registration in radiotherapy. Med Phys.

[33] Dice L.R. (1945). Measures of the amount of ecologic association between species. Ecology.

[34] Huttenlocher D.P., Klanderman G.A., Rucklidge W.J. (1993). Comparing images using the Hausdorff distance. IEEE Trans Pattern Anal Mach Intell.

[35] Tsuchida K., Minohara S., Kusano Y. (2021). Interfractional robustness of scanning carbon ion radiotherapy for prostate cancer: an analysis based on dose distribution from daily in-room CT images. J Appl Clin Med Phys.

[36] Shortall J., Vasquez Osorio E., Cree A. (2021). Inter- and intra-fractional stability of rectal gas in pelvic cancer patients during MRIgRT. Med Phys.

[37] Nijkamp J., de Jong R., Sonke J.J. (2009). Target volume shape variation during irradiation of rectal cancer patients in supine position: comparison with prone position. Radiother Oncol.

[38] Fiorino C., Foppiano F., Franzone P. (2005). Rectal and bladder motion during conformal radiotherapy after radical prostatectomy. Radiother Oncol.

[39] Nijkamp J., de Jong R., Sonke J.J. (2009). Target volume shape variation during hypo-fractionated preoperative irradiation of rectal cancer patients. Radiother Oncol.

[40] Hoogeman M.S., Van Herk M., De Bois J. (2005). Strategies to reduce the systematic error due to tumor and rectum motion in radiotherapy of prostate cancer. Radiother Oncol.

[41] Ishikawa H., Tsuji H., Kamada T. (2006). Risk factors of late rectal bleeding after carbon ion therapy for prostate cancer. Int J Radiat Oncol Biol Phys.

[42] Maebayashi T., Ishibashi N., Aizawa T. (2017). Factors predicting late rectal disorders after radiation therapy for prostate cancer. Chin Med J.

[43] Akimoto T., Muramatsu H., Takahashi M. (2004). Rectal bleeding after hypofractionated radiotherapy for prostate cancer: correlation between clinical and dosimetric parameters and the incidence of grade 2 or worse rectal bleeding. Int J Radiat Oncol Biol Phys.

[44] Sveistrup J., af Rosenschöld P.M., Deasy J.O. (2014). Improvement in toxicity in high risk prostate cancer patients treated with image-guided intensity-modulated radiotherapy compared to 3D conformal radiotherapy without daily image guidance. Radiat Oncol.

[45] Coen J.J., Bae K., Zietman A.L. (2011). Acute and late toxicity after dose escalation to 82 GyE using conformal proton radiation for localized prostate cancer: initial report of American College of Radiology Phase II study 03-12. Int J Radiat Oncol Biol Phys.

[46] Shin H.B., Kim C., Han M.C. (2023). Dosimetric comparison of robust angles in carbon-ion radiation therapy for prostate cancer. Front Oncol.

[47] Wang W., Huang Z., Sheng Y. (2020). RBE-weighted dose conversions for carbon ionradiotherapy between microdosimetric kinetic model and local effect model for the targets and organs at risk in prostate carcinoma. Radiother Oncol.

[48] Mori S., Zenklusen S., Knopf A.C. (2013). Current status and future prospects of multi-dimensional image-guided particle therapy. Radiol Phys Technol.

[49] Landry G., Hua C. ho (2018). Current state and future applications of radiological image guidance for particle therapy. Med Phys.

[50] Mori S., Hirai R., Sakata Y. (2023). Deep neural network-based synthetic image digital fluoroscopy using digitally reconstructed tomography. Phys Eng Sci Med.

[51] Kumakiri T., Mori S., Mori Y. (2023). Real-time deep neural network-based automatic bowel gas segmentation on X-ray images for particle beam treatment. Phys Eng Sci Med.

[52] Papanikolaou N., Battista J.J., Boyer A.L. (2004).

