# Supplementary figures and images for: Rectal Gas-Induced Dose Changes in Carbon Ion Radiation Therapy for Prostate Cancer: An In Silico Study
Source: Int J Part Ther. 2024 Nov 26;15:100637. doi: 10.1016/j.ijpt.2024.100637 (PMC11697597; doi:10.1016/j.ijpt.2024.100637)

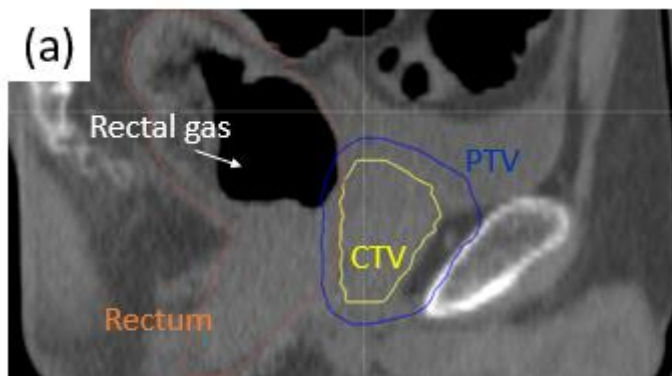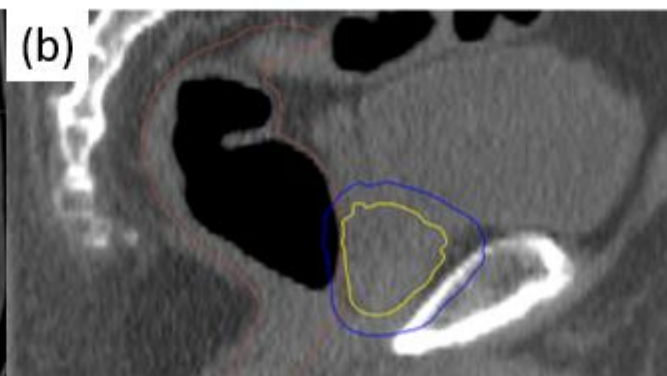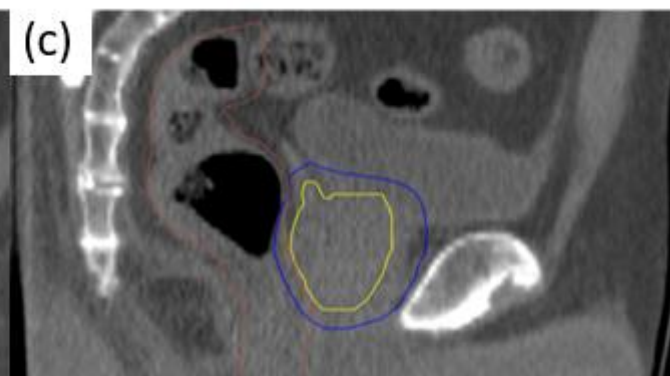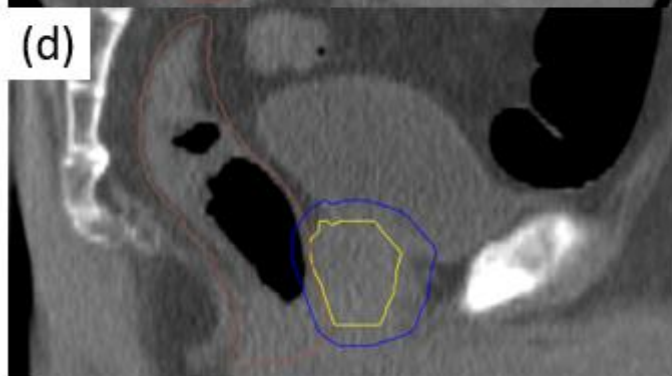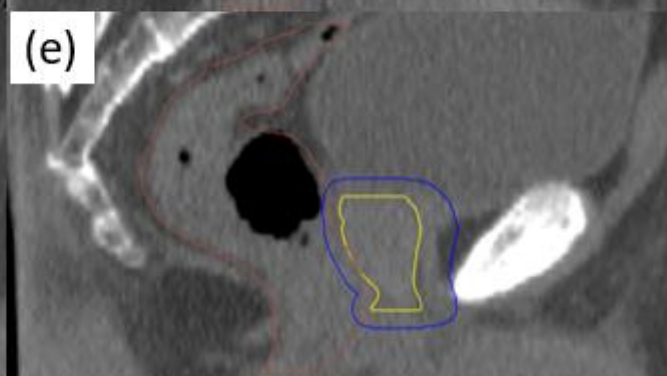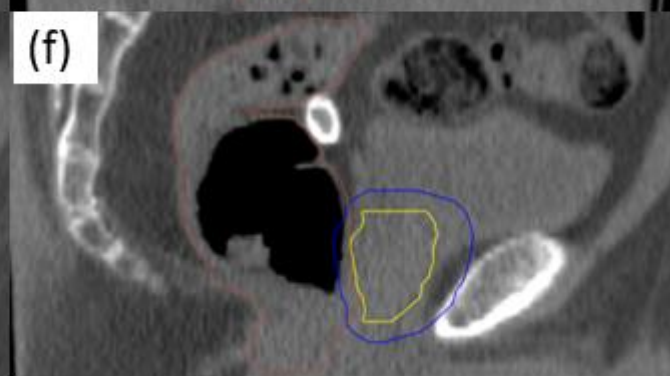

Supplement: Supplementary Figure 1 — Rectal gas location. Abbreviations: gasCT: CT of remaining bulky rectal gas. (a), (b), (c), (d), (e), and (f) show the sagittal plane of the gasCT for patients 1, 3, 4, 11, 12, and 17, respectively. CTV is outlined in yellow, PTV in dark blue, and rectum in brown. Rectal gas was mainly located superior to the CTV and rectum. [file mmc1.pdf]
