# Supplementary material for: Rectal Gas-Induced Dose Changes in Carbon Ion Radiation Therapy for Prostate Cancer: An In Silico Study
Source: Int J Part Ther. 2024 Nov 26;15:100637. doi: 10.1016/j.ijpt.2024.100637 (PMC11697597; doi:10.1016/j.ijpt.2024.100637)
Supplement: Supplementary file 2 — Supplementary material [file mmc2.docx]

Supplementary Table 1. DVH parameters with changed residual rectal gas fractions up to 12 fx

| DVH parameter | pCT (0 fx) | 1 fx | 2 fx | 3 fx |
| --- | --- | --- | --- | --- |
| CTV Dmin (Gy) | 48.5 ± 0.2 (48.0–49.0) | 48.6 ± 0.2 (48.0–49.1) | 48.5 ± 0.3 (47.9–49.2) | 48.6 ± 0.4 (47.7–49.4) |
| CTV D99.5% (Gy) | 50.8 ± 0.1 (50.7–51.0) | 50.8 ± 0.1 (50.6–51.0) | 50.8 ± 0.1 (50.5–51.0) | 50.7 ± 0.2 (50.4–51.1) |
| Rectum V95% (cc) | 0.2 ± 0.1 (0.1–0.4) | 0.3 ± 0.1 (0.1–0.4) | 0.3 ± 0.1 (0.1–0.5) | 0.3 ± 0.1 (0.1–0.6) |
| Rectum V80% (cc) | 2.8 ± 0.3 (2.1–3.4) | 2.8 ± 0.3 (2.1–3.4) | 2.8 ± 0.3 (2.1–3.4) | 2.8 ± 0.3 (2.1–3.5) |
| RW V95% (cc) | 0.2 ± 0.1 (0.1–0.4) | 0.2 ± 0.1 (0.1–0.4) | 0.3 ± 0.1 (0.1–0.4) | 0.3 ± 0.1 (0.1–0.5) |
| RW V80% (cc) | 1.4 ± 0.1 (1.2–1.7) | 1.4 ± 0.1 (1.2–1.7) | 1.4 ± 0.1 (1.2–1.7) | 1.4 ± 0.1 (1.2–1.7) |
| DVH parameter |  | 4 fx | 5 fx | 6 fx |
| CTV Dmin (Gy) |  | 48.6 ± 0.5 (47.5–49.6) | 48.5 ± 0.6 (47.3–49.8) | 48.5 ± 0.7 (47.0–50.0) |
| CTV D99.5% (Gy) |  | 50.7 ± 0.2 (50.2–51.1) | 50.6 ± 0.2 (50.0–51.2) | 50.5 ± 0.3 (49.9–51.2) |
| Rectum V95% (cc) |  | 0.4 ± 0.1 (0.1–0.6) * | 0.5 ± 0.1 (0.2–0.7) * | 0.6 ± 0.1 (0.2–0.9) * |
| Rectum V80% (cc) |  | 2.9 ± 0.3 (2.2–3.7) | 3.0 ± 0.3 (2.2–3.8) | 3.2 ± 0.4 (2.3–4.1) |
| RW V95% (cc) |  | 0.3 ± 0.1 (0.1–0.5) * | 0.4 ± 0.1 (0.2–0.6) * | 0.5 ± 0.1 (0.2–0.7) * |
| RW V80% (cc) |  | 1.5 ± 0.1 (1.2–1.8) | 1.5 ± 0.1 (1.2–1.8) | 1.5 ± 0.1 (1.2–1.9) |
| DVH parameter |  | 7 fx | 8 fx | 9 fx |
| CTV Dmin (Gy) |  | 48.5 ± 0.8 (46.8–50.2) | 48.4 ± 0.9 (46.5–50.4) | 48.4 ± 1.0 (46.2–50.6) |
| CTV D99.5% (Gy) |  | 50.5 ± 0.3 (49.7–51.3) | 50.4 ± 0.4 (49.5–51.3) | 50.3 ± 0.4 (49.3–51.3) |
| Rectum V95% (cc) |  | 0.7 ± 0.2 (0.3–1.1) * | 1.0 ± 0.2 (0.5–1.4) * | 1.2 ± 0.3 (0.6–1.8) * |
| Rectum V80% (cc) |  | 3.5 ± 0.4 (2.5–4.4) | 3.8 ± 0.5 (2.7–4.8) * | 4.3 ± 0.6 (2.9–5.6) * |
| RW V95% (cc) |  | 0.6 ± 0.1 (0.3–0.9) * | 0.7 ± 0.1 (0.4–1.0) * | 0.8 ± 0.1 (0.4–1.1) * |
| RW V80% (cc) |  | 1.6 ± 0.2 (1.2–1.9) * | 1.7 ± 0.2 (1.3–2.0) * | 1.8 ± 0.2 (1.4–2.2) * |
| DVH parameter |  | 10 fx | 11 fx | 12 fx |
| CTV Dmin (Gy) |  | 48.3 ± 1.1 (45.9–50.7) | 48.2 ± 1.2 (45.6–50.8) | 48.1 ± 1.2 (45.3–50.9) |
| CTV D99.5% (Gy) |  | 50.2 ± 0.5 (49.1–51.4) | 50.1 ± 0.5 (48.9–51.4) | 50.0 ± 0.6 (48.7–51.4) |
| Rectum V95% (cc) |  | 1.5 ± 0.3 (0.8–2.2) * | 2.1 ± 0.4 (1.1–3.1) ** | 3.1 ± 0.7 (1.5–4.7) ** |
| Rectum V80% (cc) |  | 4.9 ± 0.7 (3.3–6.6) * | 5.3 ± 0.8 (3.5–7.2) * | 5.7 ± 0.9 (3.7–7.7) * |
| RW V95% (cc) |  | 0.9 ± 0.2 (0.5–1.2) * | 1.0 ± 0.2 (0.6–1.5) * | 1.2 ± 0.2 (0.7–1.7) * |
| RW V80% (cc) |  | 1.9 ± 0.2 (1.4–2.3) * | 1.9 ± 0.2 (1.5–2.4) * | 2.0 ± 0.2 (1.5–2.5) * |

Abbreviations: CTV: clinical target volume; D_min_: minimum dose; V_x%_: the volume irradiated by x% or more of the prescribed dose; D_y%_: the dose irradiated by y% or more of the volume; RW: rectal wall

^⁎^ *p* < 0.05 compared with the pCT

^⁎ ⁎^ *p* < 0.001 compared with pCT

DVH parameters with changed residual rectal gas fractions up to 12 fx, the mean value for 18 cases, and the maximum and minimum values of the 95% confidence interval of the mean.
