# Supplementary material for: Rectal Gas-Induced Dose Changes in Carbon Ion Radiation Therapy for Prostate Cancer: An In Silico Study
Source: Int J Part Ther. 2024 Nov 26;15:100637. doi: 10.1016/j.ijpt.2024.100637 (PMC11697597; doi:10.1016/j.ijpt.2024.100637)
Supplement: Supplementary file 3 — Supplementary material [file mmc3.docx]

**Appendix**_C Additional study

**Purpose**

This additional study aimed to evaluate the reproducibility of the amount and location of rectal gas in patients with prostate cancer.

**Methods**

For this additional study, we utilized cone beam computed tomography (CBCT) data from 35 treatment fractions of 40 prostate cancer patients who underwent X-ray therapy at our hospital between June 2011 and June 2012. The eligibility criteria were as follows: rectal gas volume (≥2 cc) in ≥5 fractions, and the absence of severe artifacts. Fifteen patients met these criteria. On the CBCT scans, the rectum was outlined in the same rule as in the original study, and rectal gas was defined as any area with attenuation values of -300 HU or lower within that region.

Rectal Gas Volume: The volume of rectal gas across 5 fractions was calculated, and the mean and standard deviation were calculated. The degree of variation in the standard deviation relative to the mean was then assessed.

Rectal gas location: The rectum was divided into three sections (superior, middle, and inferior), and the rectal gas location was evaluated based on its location within these sections. Addithionaly, the rectal gas location was further measured using the height of the tailbone as a reference.

**Results and Discussion**

Rectal Gas Volume: The mean ± standard deviation of rectal gas across 5 fractions for 15 patients was 16.6 ± 8.3 cc. On a per-patient basis, the standard deviation was 2.9 cc (mean) and 2.8 cc (median). Variations in rectal gas volume between fractions ranged from 12% to 20%. In summary, rectal gas volume remained relatively consistent within individual patients.

Rectal gas location: The location of rectal gas in 75 CBCT scans from 15 patients across 5 fractions was evaluated. The results showed that 85.3% of rectal gas was located in the superior section, 9.3% in the superior and middle sections, 5.3% in the middle section, and 0% in the posterior section. In most cases, rectal gas extended from the superior to the middle section, with location differences within 5 mm across fractions. This indicated that the location of rectal gas within individual patients was reproducible, and similar trends were observed in many patients. Other studies have also reported that rectal gas tends to occur in consistent locations^1^, and our findings align with these results.

**Conclusion**

The findings suggest that the volume and location of rectal gas are reproducible within individual patients.

**Reference**

1. Shortall, J.; Vasquez Osorio, E.; Cree, A.; *et al.* Inter- and intra-fractional stability of rectal gas in pelvic cancer patients during MRIgRT. *Med Phys* 48(1):414–26; 2021. Doi: 10.1002/mp.14586.
